# *Pogo* transposons provide tools to restrict cancer growth

**DOI:** 10.1002/1878-0261.13801

**Published:** 2025-01-15

**Authors:** Elina Zueva, Marianne Burbage

**Affiliations:** ^1^ Institut Curie, Inserm U932 – Immunity and Cancer Paris France; ^2^ PSL Research University Paris France

**Keywords:** cancer growth, DNA transposons, ribosome, Tumor suppressor

## Abstract

Transposable elements provide material for novel gene formation. In particular, DNA transposons have contributed several essential genes involved in various physiological or pathological conditions. Here, we discuss recent findings by Tu et al. in *Molecular Cell* that identify *Pogo* transposon‐derived gene POGK as tumor suppressor in triple‐negative breast cancer (TNBC) by regulating ribosome biogenesis and restricting cell growth. An isoform‐switch in TNBC results in the loss of POGK capacity to recruit the epigenetic corepressor TRIM28 and to exert its repressive functions. These findings shed light on the potential for TE‐derived genes in providing new therapeutic opportunities for highly malignant TNBC.

## Graphical abstract

Here, we discuss findings by Tu et al. showing that POGK, a gene derived from the *pogo* DNA transposon, has tumor‐suppressor functions in triple‐negative breast cancer. POGK interacts with the corepressor TRIM28 to inactivate gene targets, including several ribosomal genes. POGK function is regulated by a switch between the full‐length isoform and a shorter one devoid of repressive functions.

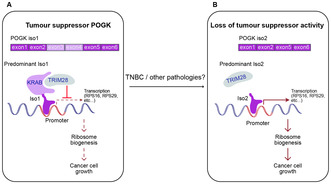

**Abbreviations**
KRAB: Krüppel‐associated box
TCGA: The Cancer Genome Atlas
TE: transposable element
TEG: transposable‐element‐derived gene
TNBC: triple‐negative breast cancer

Transposable elements (TEs) constitute a significant portion of eukaryotic genomes. They are classified into two main categories. Class I retrotransposons move via an RNA intermediate using a copy‐and‐paste mechanism, while Class II DNA transposons use DNA intermediates through “cut‐and‐paste” or a “peel‐and‐paste” mechanisms [1]. Throughout evolutionary history, the parasitic propagation of TEs has profoundly reshaped genomic architecture. Coevolving with host genomes, TEs have contributed a wide array of regulatory elements such as promoters, enhancers, organizers of 3D genomic architecture, and drivers of novel splice isoforms, which occasionally encode proteins with altered cellular functions [2, 3, 4].

Recent technological advances have revealed the remarkable ability of TEs to contribute to the emergence of novel functional genes, with estimates ranging from dozens to thousands [5]. The most striking examples include the ARC gene encoding a repurposed Gag protein derived from a Ty3/Gypsy retrotransposon and playing a critical role in synaptic plasticity [6], and the Syncytin, a retroviral envelope protein that is essential for placental morphogenesis [7]. Interestingly, DNA transposons contribute a disproportionately larger number of TE‐derived genes than retrotransposons. These elements encode transposase enzymes that recognize specific nucleotide sequences within the cognate transposons, enabling their mobilization. Both the DNA‐binding and catalytic domains of transposases can be captured for cellular functions. Transposase capture likely contributed to the development of the adaptive immune system in jawed vertebrates. The V(D)J recombination process, which generates virtually limitless diversity in antibodies and T‐cell receptors, relies on RAG1/2 recombinases. They catalyze DNA cutting and joining through chemistry closely related to DNA transposases, with the RAG1 sequence showing evolutionary links to enzymes of *Transib* DNA transposons [8], suggesting that *Transib*‐like elements gave rise to this groundbreaking evolutionary innovation. In this example, the transposase retains its original function; however, a more common evolutionary outcome is the creation of proteins with entirely novel functions. For instance, the transposase domains of *pogo* transposons, part of the Tc1/mariner superfamily that has invaded nearly all animal taxa, have been repeatedly domesticated for novel functions. In mammals, they gave rise to centromere‐associated protein CENPB, which binds α‐satellite repeats in a sequence‐specific way to facilitate kinetochore formation [9]. The fusion of transposase domains with host ZNF or Krüppel‐associated box (KRAB) domains led to the emergence of the POGZ and POGK proteins, respectively [5].

Change in expression of *pogo*‐derived genes is associated with various pathologies. For example, disruption of POGZ expression is implicated in autism and intellectual disability [10, 11]. CENPB is aberrantly expressed in several cancers, including lung and colorectal cancers [12, 13]. In a recent issue of *Molecular Cell*, Tu et al. describe a tumor‐suppressor function of POGK in triple‐negative breast cancer (TNBC) [14], the most aggressive subtype with very few known therapeutic targets. They analyzed a group of transposable‐element‐derived genes (TEGs) across diverse cancer cohorts from the Cancer Genome Atlas (TCGA), identifying POGK as the top‐altered TEG transcript, most significantly dysregulated in breast cancers. Immunohistochemical analysis revealed reduced POGK expression in breast cancer tissues compared to normal samples, with a marked decrease in TNBC relative to non‐TNBC subtypes. Functional assays demonstrated that POGK overexpression impaired TNBC cell growth *in vitro* and in both immune‐competent and immune‐compromised mice. This growth suppression was accompanied by a significant reduction in ribosomal gene transcription and ribosome biogenesis. The authors showed that the KRAB domain of POGK interacts with the global transcriptional co‐repressor TRIM28 [15] (Fig. 1A). This complex specifically binds to DNA to repress gene expression, particularly the ribosomal genes RPS16 and RPS29. Notably, the ability of POGK to suppress cell growth relies on this KRAB–TRIM28 interaction, as a KRAB domain mutant was unable to inhibit cell growth. Mechanistically, the study revealed that POGK downregulation in TNBC likely results from isoform switching. In TNBC cells, the full‐length POGK isoform (iso1, Fig. 1A) is replaced by a shorter isoform (iso2, Fig. 1B) lacking the KRAB domain. Although iso2 retains the same DNA‐binding motif as iso1, it fails to recruit TRIM28, rendering it incapable of repressing RPS16 and RPS29 transcription. Furthermore, DOX‐inducible expression of iso2, unlike iso1, did not impair TNBC cell growth either *in vitro* or *in vivo* (Fig. 1).

**Fig. 1:**
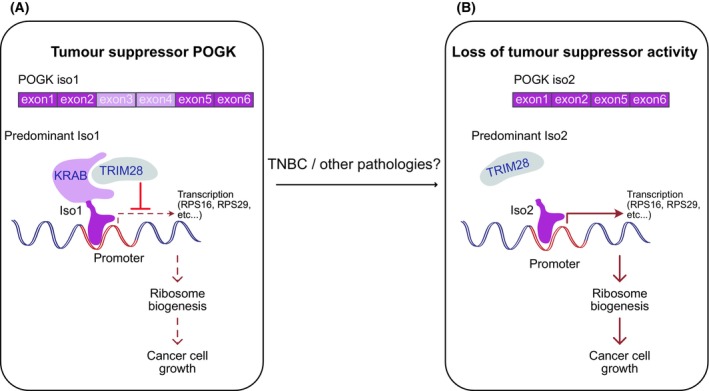
In a recent issue of *Molecular Cell*, Tu et al. show that the *Pogo* transposon‐derived gene POGK acts as a tumor‐suppressor in triple‐negative breast cancer (TNBC). The mechanism they propose relies on an isoform switch between a full‐length POGK version (Iso1, panel A) that has a KRAB domain and a shorter one (Iso2, panel B) that lacks the KRAB domain. (A) POGK Iso1 binds to specific sites on the DNA and recruits the TRIM28, an adapter protein inducing epigenetic silencing. (B) POGK Iso2 harbors the same DNA‐binding motif but is unable to recruit the repressive machinery. POGK target genes include numerous genes involved in ribosomal biogenesis, in particular Rps16 and Rps29. Overexpression of POGK Iso1, but not Iso2, results in reduced ribosome formation and severely hinders growth of cancer cells *in vitro* and *in vivo*. This paper provides the first evidence of a TE‐derived gene exapted as a tumor suppressor.

In conclusion, the study provides compelling insights into the mechanism of action of POGK, highlighting how its isoform switching undermines cancer defense mechanisms. The discovery that reduced POGK expression in TNBC arises from a shift from a full‐length isoform to a shorter one is particularly interesting. Identifying the molecular players that regulate this isoform balance will be critical for developing therapeutic strategies to restore the tumor‐suppressive POGK iso1 expression. Splicing is a highly intricate process involving core spliceosome components and cofactors that promote or repress spliceosome assembly. Recent studies suggest that the deregulation of splicing‐related proteins is a primary driver of altered splicing landscapes in cancer [16, 17]. This raises an important question about the identity of splicing factors that induce the POGK isoform switch. TNBC, particularly its glycolytic subtype MPS2, associated with higher tumor grade, exhibits profound dysregulation of splicing machinery proteins [18]. This makes TNBC potentially vulnerable to splicing‐targeting pharmacological interventions. Indeed, modulation of specific splicing‐related genes has been shown to induce TNBC cell death [19, 20, 21]. With the growing focus on pharmacological inhibition of splicing factors [22, 23] and on isoform switching drugs [24], correcting POGK isoform imbalance in TNBC offers a promising strategy to combat this aggressive cancer subtype.

The authors observed that POGK levels are both upregulated and downregulated across various cancer cohorts, raising another important question. How do the tumor‐suppressive functions of POGK‐iso1 manifest in TNBC subtypes or other tumor types, and to what extent is POGK expression predictive of patient survival or treatment response?

From a genomic perspective, Tu et al. provide the first evidence of a DNA transposon‐derived fusion protein acting as a tumor suppressor. As the investigation of TE‐derived proteins continues to advance, additional narratives will likely emerge, shedding light on these molecular chimeras born from the complex interplay between host genomes and their parasitic invaders.

## Conflict of interest

The authors declare no conflict of interest.

## Author contributions

EZ and MB both analyzed and discussed the Tu et al. paper, wrote and edited the manuscript.

## References

[1] Bourque G , Burns KH , Gehring M , Gorbunova V , Seluanov A , Hammell M , et al. Ten things you should know about transposable elements. Genome Biol. 2018;19:1–12. 30454069 10.1186/s13059-018-1577-z PMC6240941

[2] Fueyo R , Judd J , Feschotte C , Wysocka J . Roles of transposable elements in the regulation of mammalian transcription. Nat Rev Mol Cell Biol. 2022;23:481–497. 35228718 10.1038/s41580-022-00457-y PMC10470143

[3] Arribas YA , Baudon B , Rotival M , Suárez G , Bonté PE , Casas V , et al. Transposable element exonization generates a reservoir of evolving and functional protein isoforms. Cell. 2024;187:7603–7620. 39667937 10.1016/j.cell.2024.11.011

[4] Pasquesi GIM , Allen H , Ivancevic A , Barbachano‐Guerrero A , Joyner O , Guo K , et al. Regulation of human interferon signaling by transposon exonization. Cell. 2024;187:7621–7636.e19. 10.1016/j.cell.2024.11.016 39672162 PMC11682929

[5] Feschotte C , Pritham EJ . DNA transposons and the evolution of eukaryotic genomes. Annu Rev Genet. 2007;41:331–368. 18076328 10.1146/annurev.genet.40.110405.090448 PMC2167627

[6] Zhang W , Wu J , Ward MD , Yang S , Chuang YA , Xiao M , et al. Structural basis of arc binding to synaptic proteins: implications for cognitive disease. Neuron. 2015;86:490–500. 25864631 10.1016/j.neuron.2015.03.030 PMC4409568

[7] Mi S , Lee X , Li XP , Veldman GM , Finnerty H , Racie L , et al. Syncytin is a captive retroviral envelope protein involved in human placental morphogenesis. Nature. 2000;403:785–789. 10693809 10.1038/35001608

[8] Kapitonov VV , Jurka J . RAG1 core and V(D)J recombination signal sequences were derived from Transib transposons. PLoS Biol. 2005;3:e181. 15898832 10.1371/journal.pbio.0030181 PMC1131882

[9] Fachinetti D , Diego Folco H , Nechemia‐Arbely Y , Valente LP , Nguyen K , Wong AJ , et al. A two‐step mechanism for epigenetic specification of centromere identity and function. Nat Cell Biol. 2013;15:1056–1066. 23873148 10.1038/ncb2805 PMC4418506

[10] White J , Beck CR , Harel T , Posey JE , Jhangiani SN , Tang S , et al. POGZ truncating alleles cause syndromic intellectual disability. Genome Med. 2016;8:1–11. 26739615 10.1186/s13073-015-0253-0 PMC4702300

[11] Stessman HAF , Willemsen MH , Fenckova M , Penn O , Hoischen A , Xiong B , et al. Disruption of POGZ is associated with intellectual disability and autism spectrum disorders. Am J Hum Genet. 2016;98:541–552. 26942287 10.1016/j.ajhg.2016.02.004 PMC4890241

[12] Briasoulis E , Kamposioras K , Tzovaras V , Pafitanis G , Kostoula A , Mavridis A , et al. CENP‐B specific anti‐centromere autoantibodies heralding small‐cell lung cancer: a case study and review of the literature. Lung Cancer. 2008;60:302–306. 17980453 10.1016/j.lungcan.2007.09.014

[13] Wallander K , Thutkawkorapin J , Sahlin E , Lindblom A , Lagerstedt‐Robinson K . Massive parallel sequencing in a family with rectal cancer. Hered Cancer Clin Pract. 2021;19:23. 33827643 10.1186/s13053-021-00181-2 PMC8028209

[14] Tu Z , Bassal MA , Bell GW , Zhang Y , Hu Y , Quintana LM , et al. Tumor‐suppressive activities for pogo transposable element derived with KRAB domain via ribosome biogenesis restriction. Mol Cell. 2024;84:4209–4223.e6. 39481384 10.1016/j.molcel.2024.09.025

[15] Czerwińska P , Mazurek S , Wiznerowicz M . The complexity of TRIM28 contribution to cancer. J Biomed Sci. 2017;24:1–14. 28851455 10.1186/s12929-017-0374-4 PMC5574234

[16] Papasaikas P , Tejedor JR , Vigevani L , Valcárcel J . Functional splicing network reveals extensive regulatory potential of the core spliceosomal machinery. Mol Cell. 2015;57:7–22. 25482510 10.1016/j.molcel.2014.10.030

[17] Kahles A , Lehmann KV , Toussaint NC , Hüser M , Stark SG , Sachsenberg T , et al. Comprehensive analysis of alternative splicing across tumors from 8,705 patients. Cancer Cell. 2018;34:211–224.e6. 30078747 10.1016/j.ccell.2018.07.001 PMC9844097

[18] Yang W , Hong L , Guo L , Wang Y , Han X , Han B , et al. Targeting SNRNP200‐induced splicing dysregulation offers an immunotherapy opportunity for glycolytic triple‐negative breast cancer. Cell Discov. 2024;10:96. 39285160 10.1038/s41421-024-00715-7 PMC11405407

[19] An J , Luo Z , An W , Cao D , Ma J , Liu Z . Identification of spliceosome components pivotal to breast cancer survival. RNA Biol. 2021;18:833–842. 32965163 10.1080/15476286.2020.1822636 PMC8081035

[20] Koedoot E , van Steijn E , Vermeer M , González‐Prieto R , Vertegaal ACO , Martens JWM , et al. Splicing factors control triple‐negative breast cancer cell mitosis through SUN2 interaction and sororin intron retention. J Exp Clin Cancer Res. 2021;40:82. 33648524 10.1186/s13046-021-01863-4 PMC7919097

[21] Lu X‐X , Yang WX , Pei YC , Luo H , Li XG , Wang YJ , et al. An in vivo CRISPR screen identifies that SNRPC promotes triple‐negative breast cancer progression. Cancer Res. 2023;83:2000–2015. 37057875 10.1158/0008-5472.CAN-22-0536

[22] Kashyap MK , Kumar D , Villa R , la Clair JJ , Benner C , Sasik R , et al. Targeting the spliceosome in chronic lymphocytic leukemia with the macrolides FD‐895 and pladienolide‐B. Haematologica. 2015;100:945–954. 25862704 10.3324/haematol.2014.122069 PMC4486229

[23] Sato M , Muguruma N , Nakagawa T , Okamoto K , Kimura T , Kitamura S , et al. High antitumor activity of pladienolide B and its derivative in gastric cancer. Cancer Sci. 2014;105:110–116. 24635824 10.1111/cas.12317 PMC4317874

[24] Jbara A , Siegfried Z , Karni R . Splice‐switching as cancer therapy. Curr Opin Pharmacol. 2021;59:140–148. 34217945 10.1016/j.coph.2021.05.008

